# Infant Dyschezia Is Associated With Increased Risk of Functional Constipation in Childhood: A Case–Control Study

**DOI:** 10.1155/ijpe/6410431

**Published:** 2026-07-02

**Authors:** Mahbod Kaveh, Arman Malekiantaghi, Hamideh Mohammadi, Hosein Shabani-Mirzaee, Kambiz Eftekhari

**Affiliations:** ^1^ Department of Neonatology, Bahrami Children′s Hospital, School of Medicine, Tehran University of Medical Sciences, Tehran, Iran, tums.ac.ir; ^2^ Department of Pediatric Gastroenterology, Bahrami Children′s Hospital, School of Medicine, Tehran University of Medical Sciences, Tehran, Iran, tums.ac.ir; ^3^ Department of Pediatric, Bahrami Children′s Hospital, School of Medicine, Tehran University of Medical Sciences, Tehran, Iran, tums.ac.ir; ^4^ Pediatric Gastroenterology and Hepatology Research Center, Department of Pediatric Gastroenterology, Bahrami Children′s Hospital, School of Medicine, Tehran University of Medical Sciences, Tehran, Iran, tums.ac.ir

**Keywords:** functional constipation, infant dyschezia, pediatric gastrointestinal disorders, Rome IV criteria

## Abstract

**Background:**

Functional constipation (FC) frequently occurs in children and leads to notable discomfort as well as substantial healthcare costs. Infant dyschezia (ID) is characterized by excessive straining and crying before passing soft stools in infants and is traditionally considered benign and self‐limiting. However, emerging evidence suggests a possible link between ID and later chronic bowel dysfunction. The objective of this work was to examine whether a history of ID is linked to the later emergence of FC during childhood.

**Methods:**

We recruited 150 children for this case–control investigation, including 90 individuals diagnosed with FC and 60 healthy controls, all from a tertiary pediatric outpatient clinic. Diagnoses were based on Rome IV criteria. Data on demographics, infant feeding, family history of constipation, and history of ID were collected through structured interviews. We applied logistic regression analysis to determine which factors independently predicted FC.

**Results:**

Children with FC were significantly more likely than controls to have a history of ID (66.7% vs. 40.0%, *p* = 0.001). On multivariate analysis, ID remained independently associated with FC (OR = 2.75; 95% CI: 1.34–5.64; *p* = 0.006). Breastfeeding during infancy (either exclusive or partial) showed a protective effect (OR = 0.22; 95% CI: 0.09–0.56; *p* = 0.002). The FC group contained more boys (54.4% vs. 35.0%), but this difference was not statistically significant (*p* = 0.057).

**Conclusion:**

These results indicate that ID could serve as an independent risk factor for childhood FC. Early recognition and parental guidance are essential to prevent mismanagement and long‐term morbidity. Longitudinal studies are recommended to confirm causality and inform preventive strategies.

## 1. Introduction

Functional constipation (FC) represents a frequently diagnosed gastrointestinal condition in pediatric populations and is responsible for a large number of referrals to pediatric gastroenterology services globally [1]. Its prevalence ranges from 0.5% to 30%, depending on the population studied and the diagnostic criteria used [2]. The Rome IV system defines FC based on the presence of several clinical features, including rare bowel motions, holding back stool, discomfort during defecation, or passing very large stools, all occurring without any underlying structural problem [2]. Etiological factors include dietary habits, behavioral patterns, delayed toilet training, and psychosocial influences [3].

Infant dyschezia (ID) is a functional bowel condition that occurs in infants under 9 months of age. These infants have repeated episodes of straining and crying lasting a minimum of 10 min prior to the passage of soft stools, and they are otherwise healthy with no underlying organic disease [4]. Although ID is generally benign and self‐limiting, it is often misunderstood by caregivers and healthcare providers, leading to unnecessary interventions [4]. The prevalence of ID varies with age and population. Studies report prevalence rates ranging from 2.4% to 22.1% during infancy, with higher rates in the first weeks of life [4, 5].

Although both ID and FC are functional gastrointestinal disorders (FGIDs), their relationship has not been fully elucidated. Most studies have examined their epidemiology separately, and limited evidence suggests a possible link between ID and later FC [6, 7]. Variability in population characteristics, diagnostic criteria (Rome III vs. Rome IV), and cultural perceptions of normal infant defecation may contribute to inconsistent findings. Given the chronic nature and burden of FC, early identification of predictors like ID could enable timely intervention and reduce long‐term complications.

The present study sought to investigate whether ID is associated with FC in children referred to Bahrami Hospital in Tehran, Iran. We hypothesize that ID may serve as an independent predictor for the development of FC during childhood.

## 2. Methods

### 2.1. Study Design

We employed a case–control design to explore whether a relationship exists between ID and FC in the pediatric population. This case–control design enabled us to compare children with constipation (cases) to healthy children without constipation (controls), thereby helping to identify possible risk factors and associations.

### 2.2. Setting and Duration

This research took place in the pediatric gastroenterology outpatient department of Bahrami Hospital, located in Tehran, Iran. The process of gathering data extended across 2 years, specifically from January 2021 to December 2022.

### 2.3. Participants

#### 2.3.1. Inclusion Criteria

Only otherwise healthy children presenting with constipation and meeting the Rome IV diagnostic standards for ID or FC were included in the study. Before they entered the study, we obtained written informed consent from the parents or legal guardians of every child and gave them a complete explanation of the study′s purposes and procedures.

#### 2.3.2. Exclusion Criteria

We excluded children with organic constipation causes, including Hirschsprung′s disease and structural gut abnormalities. Moreover, those with irritable bowel syndrome (diagnosed using Rome IV criteria), metabolic disorders (e.g., hypothyroidism), neurological impairments (including intellectual disability), history of gastrointestinal surgeries (e.g., bowel resection), and those on medications affecting gastrointestinal motility (e.g., cisapride, metoclopramide, erythromycin, and loperamide) were also excluded. These exclusions were intended to reduce confounding factors that could influence bowel habits independently of ID.

### 2.4. Variables and Data Collection

A structured form, created specifically for this project, was used to gather demographic and clinical information. This instrument was designed according to the Rome IV criteria and comprised sections covering demographics, feeding practices, bowel habits, and clinical features of ID and FC. All final diagnoses were verified by board‐certified pediatric gastroenterologists following a thorough clinical assessment based on the Rome IV definitions [2, 4]. The structured data collection form used in this study is provided as Supporting Information S1. We made the diagnoses of ID and FC according to the Rome IV criteria, based on clinical assessment. We also documented each child′s family history of chronic constipation (meaning chronic constipation in a parent or sibling) along with any concomitant medical conditions. Feeding type during infancy was categorized as exclusive breastfeeding, formula feeding, or mixed feeding. Additionally, we recorded whether the child consumed more than 500 cc of milk per day (including breast milk, formula, or cow′s milk) at the time of the study. Key clinical features, such as bowel habits, stool characteristics, symptom duration, and physical examination findings, were carefully assessed. Variables were analyzed as categorical or continuous depending on the nature of the data.

### 2.5. Sample Size

We calculated the sample size to achieve adequate statistical power for detecting meaningful differences between the groups. Using a 95% confidence level and 80% power, and based on earlier constipation prevalence figures (4.9% among cases and 0.3% among controls according to Kramer et al. [7]), we estimated that 150 participants (75 per group) would be necessary. However, during the enrollment period, we observed more eligible children with FC and fewer healthy controls willing to take part, which led to a final distribution of 90 cases and 60 controls. This group size discrepancy was taken into account in the statistical analyses.

### 2.6. Data Collection Procedure

A structured questionnaire was administered to parents or guardians to gather the study data. The questionnaire collected key data points: demographic characteristics (age, sex, and infant feeding type: exclusive breastfeeding, formula, or mixed), daily milk volume (above or below 500 cc), and snacking patterns—defined as regular intake of processed snack items such as chips, cookies, crackers, or fast food between main meals. Family history of chronic constipation and any underlying medical conditions were also gathered. Detailed bowel habits were recorded, focusing on stool characteristics and defecation behavior during infancy and childhood. For ID, parents were asked to recall whether the episodes of straining and crying lasted less than 10 min, approximately 10 min, or more than 10 min, based on the Rome IV criteria. This was an approximate estimation based on parental recall, not real‐time prospective observation. We acknowledge that this approach is subject to recall bias. Diagnosis was confirmed by pediatric gastroenterologists through comprehensive clinical evaluation, incorporating history‐taking, physical examination, and application of the Rome IV criteria. Clinical findings were further validated via direct discussions with parents to confirm symptoms and exclude alternative diagnoses.

## 3. Statistical Analysis

We carried out the statistical analyses with SPSS software (Version 25). The Kolmogorov–Smirnov test was used to check the normality of continuous variables. For continuous data, results are given as mean ± standard deviation (SD) or median (interquartile range), whereas categorical variables are shown as frequencies and percentages. To compare categorical data, we applied either the chi‐square or Fisher′s exact test. For continuous variables, the Student′s t‐test was used when the data followed a normal distribution; if not, the Mann–Whitney *U* test was applied. Logistic regression was performed to identify independent predictors of childhood constipation, incorporating variables that showed significant associations in univariate analyses. A *p* value below 0.05 was regarded as statistically significant.

### 3.1. Ethical Considerations

We carried out this study according to the principles of the Declaration of Helsinki. The Ethics Committee at Children′s Medical Center Hospital, Tehran University of Medical Sciences approved the study protocol (Approval Code: IR.TUMS.CHMC.REC.1400.144). Written informed consent was obtained from the parents or legal guardians of all participating children before they entered the study. All medical evaluations were given at no cost. Families were told that they could withdraw their child from the study at any time without any effect on their child′s usual medical care. The confidentiality and privacy of every participant were carefully protected throughout the research. The results are intended to help advance pediatric health outcomes, especially in the area of childhood digestive disorders.

## 4. Results

The study included 150 children in total: 90 with FC and 60 healthy individuals serving as controls. The fact that the groups were not equal in size (90 vs. 60) was accounted for in all statistical analyses. The participants had a mean age of 5.10 ± 3.25 years, and there was no significant difference in age between the two groups (*p* = 0.217) (Table 1).

**Table 1:** Demographic and clinical characteristics of study participants.

Variable	Constipation (*n* = 90)	Control (*n* = 60)	Total (*n* = 150)	*p*
Age (years), mean ± SD	5.11 ± 3.64	5.08 ± 2.58	5.10 ± 3.25	0.217 ^∗^
Male, *n* (%)	49 (54.4%)	21 (35.0%)	70 (46.7%)	0.019 ^∗∗^
Breastfeeding during infancy, *n* (%)	36 (40.0%)	51 (85.0%)	87 (58.0%)	< 0.001 ^∗∗^
Family history of chronic constipation, *n* (%)	50 (55.6%)	24 (40.0%)	74 (49.3%)	0.062 ^∗∗^
Infant dyschezia, *n* (%)	60 (66.7%)	24 (40.0%)	84 (56.0%)	0.001 ^∗∗^
Bowel movement frequency (infancy), mean ± SD	1.45 ± 0.65	1.50 ± 0.62	1.47 ± 0.64	0.542 ^∗^
Bowel movement frequency (childhood), mean ± SD	1.40 ± 0.98	1.47 ± 0.91	1.43 ± 0.95	0.658 ^∗^
Duration of infant dyschezia episode (minutes), mean ± SD	5.44 ± 2.08	5.28 ± 2.01	5.38 ± 2.04	0.721 ^∗^

*Note:* Single asterisk “ ^∗^” denotes Student′s t‐test; double asterisks “ ^∗∗^” denote chi‐square test. Breastfeeding during infancy refers to exclusive or partial breastfeeding in the first 6 months of life. Family history of chronic constipation was defined as a history of chronic constipation in parents or siblings.

### 4.1. Demographic Characteristics

Males constituted 46.7% (*n* = 70) of the total population. Nevertheless, the constipation group had a notably larger share of males (54.4%) than the control group (35.0%) (*p* = 0.019) (Table 1). Concerning the type of feeding during infancy, a history of breastfeeding (either exclusive or partial) was observed much more often in the control group (85.0%) than in the constipation group (40.0%) (*p* < 0.001) (Table 2).

**Table 2:** Milk feeding and snacking behaviors in constipation and control groups.

Variable	Constipation (*n* = 90)	Control (*n* = 60)	Total (*n* = 150)	*p*
Breastfeeding during infancy, *n* (%)	36 (40.0%)	51 (85.0%)	87 (58.0%)	< 0.001 ^∗∗^
Current snacking, *n* (%)	79 (87.8%)	52 (86.7%)	131 (87.3%)	0.856 ^∗∗^

*Note:* Double asterisks “ ^∗∗^” denote chi‐square test. Current snacking was defined as regular consumption of commercially prepared snack foods (e.g., chips, cookies, crackers, and fast food) between main meals at the time of the study. Breastfeeding during infancy refers to exclusive or partial breastfeeding in the first 6 months of life.

### 4.2. Clinical and Dietary Characteristics

The overall frequency of ID during infancy was 56.0%. The proportion was significantly higher, however, among constipated children (66.7%) compared with healthy controls (40.0%) (*p* = 0.001) (Table 3). The frequency of current snacking (defined as regular consumption of snack foods such as chips, cookies, and fast food between meals) was high in both groups, with 87.3% of participants reporting snacking, but no statistically significant difference was observed between the groups (*p* = 0.856) (Table 2).

**Table 3:** Logistic regression analysis of factors associated with functional constipation.

Variable	Univariate OR (95% CI)	*p*	Multivariate OR (95% CI)	*p*
Male gender	2.29 (1.15–4.55)	0.018	1.89 (0.98–3.65)	0.057
Breastfeeding during infancy	0.15 (0.07–0.33)	< 0.001	0.22 (0.09–0.56)	0.002
Infant dyschezia	2.92 (1.46–5.82)	0.002	2.75 (1.34–5.64)	0.006
Family history of chronic constipation	1.90 (0.99–3.66)	0.055	1.58 (0.78–3.19)	0.206

*Note:* Family history of chronic constipation was defined as a history of chronic constipation in parents or siblings.

Children with a family history of chronic constipation (i.e., constipation in a parent or sibling) were more common in the constipation group (55.6%) than in the control group (40.0%); however, this difference was not statistically significant (*p* = 0.062) (Table 3).

### 4.3. Quantitative Characteristics

The affected children had a mean symptom onset age of 2.05 ± 1.04 years for constipation (Table 1). The average duration of ID episodes, based on parental estimation, was 5.38 ± 2.04 min per episode. One should interpret this estimate carefully, given the risk of recall bias. The mean frequency of bowel movements during infancy and childhood was similar between the two groups (1.47 ± 0.64 and 1.43 ± 0.95 times per day, respectively).

### 4.4. Logistic Regression Analysis

In the univariate analysis, logistic regression showed that male sex (OR: 2.29; 95% CI: 1.15–4.55; *p* = 0.018), milk intake (OR: 0.15; 95% CI: 0.07–0.33; *p* < 0.001), and a history of ID (OR: 2.92; 95% CI: 1.46–5.82; *p* = 0.002) were each significantly associated with FC.

After adjusting for other variables, ID continued to be a strong independent predictor (OR: 2.75; 95% CI: 1.34–5.64; *p* = 0.006), whereas milk consumption showed a protective effect against constipation (OR: 0.22; 95% CI: 0.09–0.56; *p* = 0.002). Male sex did not remain significantly associated with FC in the multivariate model (OR = 1.89; 95% CI: 0.98–3.65; *p* = 0.057) (Table 3).

## 5. Discussion

Our findings reveal a meaningful link between ID and the later emergence of FC during childhood. Compared with children without ID, those who had experienced this condition faced almost three times the risk of subsequently developing FC. This association persisted after accounting for potential confounders including sex and current dietary habits, indicating that ID might act as a noteworthy early indicator of later gastrointestinal problems.

ID is a benign functional condition in which infants strain excessively and cry before passing soft stools [4]. Both parents and, on occasion, clinicians frequently mistake it for true constipation, leading to unnecessary medical interventions and higher levels of caregiver distress [8]. Our findings align with previous longitudinal cohort studies, which suggest that ID could potentially evolve into chronic constipation through mechanisms such as painful defecation episodes and subsequent stool withholding behavior, both of which contribute to stool hardening and the persistence of constipation [6, 9, 10].

An important finding of our study is that the protective effect of milk consumption was specifically attributable to breastfeeding. This aligns with a large body of evidence showing that breast milk promotes healthy bowel function through multiple mechanisms, including its prebiotic properties, optimal composition, and positive effects on gut microbiota [11, 12]. Our results highlight the importance of nutritional factors in gastrointestinal health. However, it is worth noting that cultural differences and parental perceptions regarding bowel habits may influence feeding practices and reporting accuracy, which could affect the interpretation of these findings [11].

In our study population, the prevalence of ID was 56%, which exceeds the figures reported in several population‐based epidemiological investigations (e.g., 3.9%–22.1% in Gatzinsky et al. [5] and 4.9% in Kramer et al. [7]). This discrepancy can be explained by several factors. First, our case–control design included a high proportion of children with FC (90 out of 150 participants), who may be more likely to have had early defecation disorders. Second, the retrospective nature of our data collection may have introduced recall bias, particularly among parents of children with FC who might recall infantile symptoms more vividly. Third, because our study took place at a tertiary referral center, parents there may be more alert to any gastrointestinal issues in their children. Finally, differences in diagnostic criteria (Rome IV vs. Rome III) and cultural perceptions of normal infant defecation may also contribute to this variability. These factors should be considered when interpreting our findings.

Our work additionally highlights how critical it is to correctly distinguish early on between ID and FC, so as to prevent unnecessary treatment and lessen parental distress. Even though the Rome IV criteria offer clear diagnostic guidance, they are still not widely used in everyday clinical practice because both healthcare providers and parents lack sufficient awareness of them [13, 14]. Improved education and dissemination of these criteria are essential to enhance both diagnosis and management.

## 6. Limitations

This study has several limitations that should be considered when interpreting the findings. Because we used a case–control design, we cannot draw causal conclusions about the relationship between ID and FC. Despite observing a significant association (OR = 2.75), this study design does not allow for causal inference. Recall bias represents a significant issue, since all ID‐related information (e.g., episode length and defecation patterns) was obtained retrospectively from parents, often several years after the infant period. Parents estimated episode duration using Rome IV categories (< 10, ~10, or > 10 min), which provides an approximation rather than exact timing. Mothers and fathers of children with FC might remember symptoms more clearly (case‐directed recall bias), possibly inflating the observed relationship. Likewise, reports of daily milk volume (> 500 cc) depended on parent memory and may lack precision. Selection bias could limit applicability, as we enrolled all subjects from just one tertiary referral center, which may include a higher proportion of severe or long‐standing constipation. Thus, our results might not extend to primary care, community‐based populations, or other geographical and cultural contexts. Although we controlled for sex, breastfeeding, and family history, we failed to assess several other potentially important confounders—including psychosocial stress, toilet training methods, dietary fiber intake, nonmilk fluid consumption, physical activity levels, and socioeconomic status. These unmeasured variables might independently affect the development of constipation. One measurement shortcoming is that we grouped all milk intake together simply as > 500 cc/day, without separating breast milk, formula, and cow′s milk—each of which has distinct effects on bowel function. The protective influence we observed was specifically linked to having been breastfed during infancy, rather than to the volume of milk being consumed at the time of the study. Although we used the Bristol Stool Form Scale (validated in children), newer scales like BITSS may be more sensitive for nontoilet‐trained infants, and the modified Bristol Scale (mBSFS‐C) may improve agreement for children. Withholding behaviors were assessed by parental report without a validated scale. The sample size imbalance (90 cases vs. 60 controls) may have affected statistical power and precision, though logistic regression remains acceptable with this distribution. Moreover, the cross‐sectional measurement of present‐day dietary habits (milk intake and snacking) restricts our ability to establish temporal sequencing; reverse causation (where dietary adjustments occur because of constipation) cannot be ruled out. Finally, because this study was conducted only in Iran, the results may not be directly transferable to other healthcare systems or cultural settings where infant feeding practices and views on defecation differ.

Future prospective cohort studies with larger, multicenter, diverse populations; comprehensive assessment of confounders; real‐time dietary diaries; age‐appropriate validated stool scales (BITSS for infants and mBSFS‐C for children); and standardized withholding behavior instruments are needed to confirm causality and inform preventive strategies. Like many case–control investigations, our study could not account for all potential confounders. Unmeasured elements, including psychosocial stressors, toilet training approaches, fiber intake, fluid consumption, and physical activity, could affect the risk of developing FC. These variables should be investigated in future prospective studies to determine whether they modify or mediate the relationship between ID and later constipation. From a practical standpoint, our results emphasize that pediatricians and primary care clinicians should look for ID during early checkups and provide parents with reassurance about its self‐limited character along with guidance on appropriate handling. Preventive strategies centered on sustaining appropriate milk intake, encouraging regular stooling patterns, and avoiding stool retention might lower the frequency of childhood constipation—a disorder associated with substantial illness burden and reduced quality of life [1, 15].

## 7. Conclusion

To sum up, our data indicate that ID represents an independent risk factor for childhood FC. While this finding highlights a potential link between early defecation patterns and later bowel dysfunction, the results should be interpreted with consideration of the study′s retrospective design and sample size. Awareness of this potential link could provide a chance for timely parent counseling and accurate diagnosis in infancy, thereby avoiding inappropriate treatment and lessening family worry. However, establishing causality and evaluating whether interventions directed at ID could decrease the risk of subsequent FC will require larger prospective cohort studies in diverse populations and healthcare settings.

## Author Contributions

Mahbod Kaveh, Kambiz Eftekhari, and Hamideh Mohammadi were involved extensively throughout all stages of the study, including conception, design, data collection, analysis, interpretation, and manuscript drafting. Arman Malekiantaghi and Hosein Shabani‐Mirzaee contributed moderately at various stages of the study and participated as coauthors. Their roles included data collection, analysis support, and manuscript preparation. Kambiz Eftekhari supervised the project, coordinated the research process, and served as the corresponding author responsible for all correspondence with the journal.

## Funding

No funding was received for this manuscript.

## Disclosure

All authors have reviewed and approved the final version of the manuscript and accept full responsibility for all parts of this work.

## Conflicts of Interest

The authors declare no conflicts of interest.

## Supporting information


**Supporting Information** Additional supporting information can be found online in the Supporting Information section. Supporting Information S1: Structured data collection form.

## Data Availability

The data underlying this study can be requested from the corresponding author. These data are not publicly accessible because of privacy and ethical considerations.
