# Supplementary material for: Infant Dyschezia Is Associated With Increased Risk of Functional Constipation in Childhood: A Case–Control Study
Source: Int J Pediatr. 2026 Jul 2;2026:6410431. doi: 10.1155/ijpe/6410431 (PMC13329262; doi:10.1155/ijpe/6410431)
Supplement: Supplementary file 1 — Supporting Information Additional supporting information can be found online in the Supporting Information section. Supporting Information S1: Structured data collection form. [file IJPE-2026-6410431-s001.docx]

**Supplementary Material 1: Structured Data Collection Form**

**Study Title:** **Infant Dyschezia is Associated with Increased Risk of Functional Constipation in Childhood: A Case-Control Study**

**Section 1: Demographic Information**

| Variable | Response |
| --- | --- |
| Child's age (years) | ......... |
| Sex | □ Male □ Female |
| Birth weight (grams) | ......... |
| Current age (years) | ......... |
| Date of interview | ......... |

**Section 2: Infant Feeding History (First 6 Months)**

| Question | Response |
| --- | --- |
| Type of feeding during infancy | □ Exclusive breastfeeding □ Exclusive formula feeding □ Mixed (breast + formula) |
| Duration of breastfeeding (months) | ......... |
| Age at introduction of formula (months) | ......... |
| Age at introduction of cow's milk (months) | ......... |

**Section 3: Current Milk Consumption (at the time of study)**

| Question | Response |
| --- | --- |
| Does the child currently consume milk? | □ Yes □ No |
| If yes, approximate daily milk intake | □ < 500 cc/day □ ≥ 500 cc/day |
| Type of milk currently consumed | □ Breast milk □ Formula □ Cow's milk □ Mixed |
| If mixed, specify: | ......... |

**Section 4: Rome IV Criteria for Infant Dyschezia (during infancy, <9 months)**

*Based on parental recall*

| Question | Response |
| --- | --- |
| Did your infant experience straining and crying before passing stool? | □ Yes □ No |
| Duration of each straining/crying episode | □ < 10 minutes □ ≥ 10 minutes |
| Stool consistency during that period | □ Soft □ Hard |
| Frequency of bowel movements during infancy (times/day) | ......... |
| Age at which these symptoms started (months) | ......... |
| Age at which these symptoms resolved (months) | ......... |

**Diagnosis of Infant Dyschezia (Rome IV criteria):**
□ Yes (all of the above: straining/crying ≥10 minutes + soft stools + age <9 months)
□ No

**Section 5: Rome IV Criteria for Functional Constipation (current)**

| Question | Response |
| --- | --- |
| Bowel movement frequency (times/week) | ......... |
| Presence of large-diameter stools that may obstruct the toilet | □ Yes □ No |
| Presence of hard stools (like rabbit pellets) | □ Yes □ No |
| Presence of stool withholding behavior | □ Yes □ No |
| Painful defecation | □ Yes □ No |
| Presence of fecal incontinence (soiling) | □ Yes □ No |
| Age at onset of constipation symptoms (years) | ......... |
| Duration of symptoms (months) | ......... |

**Diagnosis of Functional Constipation (Rome IV criteria):**
□ Yes (≥2 criteria for at least 1 month)
□ No

**Section 6: Defecation Patterns**

| Question | Infancy (0-12 months) | Childhood (current) |
| --- | --- | --- |
| Average bowel movements per day | ......... | ......... |
| Usual stool consistency | □ Soft □ Formed □ Hard | □ Soft □ Formed □ Hard |
| Straining during defecation | □ Never □ Sometimes □ Always | □ Never □ Sometimes □ Always |
| Crying before defecation | □ Never □ Sometimes □ Always | □ Never □ Sometimes □ Always |

**Section 7: Family History**

| Question | Response |
| --- | --- |
| Family history of chronic constipation | □ Yes □ No □ Unknown |
| If yes, relationship to child | □ Mother □ Father □ Sibling □ Grandparent □ Other |
| Family history of other gastrointestinal disorders | □ Yes (specify: .........) □ No |

**Section 8: Current Dietary Habits**

| Question | Response |
| --- | --- |
| Daily snacking (chips, cookies, fast food) | □ Yes □ No |
| If yes, frequency | □ 1-2 times/day □ 3-4 times/day □ >4 times/day |
| Fruit consumption | □ Daily □ Several times/weeks □ Rarely □ Never |
| Vegetable consumption | □ Daily □ Several times/weeks □ Rarely □ Never |
| Water/fluid intake (excluding milk) | □ < 4 glasses/day □ 4-6 glasses/day □ >6 glasses/day |
| Fiber-rich food consumption | □ Daily □ Several times/weeks □ Rarely □ Never |

**Section 9: Physical Activity**

| Question | Response |
| --- | --- |
| Daily physical activity (outdoor play, sports) | □ < 1 hour/day □ 1-2 hours/day □ >2 hours/day |
| Screen time (TV, tablet, phone) | □ < 1 hour/day □ 1-2 hours/day □ >2 hours/day □ >3 hours/day |

**Section 10: Psychosocial Factors (optional, based on parental report)**

| Question | Response |
| --- | --- |
| Recent stressful life events (new school, sibling birth, family conflict) | □ Yes □ No |
| If yes, specify: | ......... |
| Toilet training completed? | □ Yes (age: .... years) □ No □ Not applicable |
| Any behavioral concerns reported by parents | □ Yes (specify: .........) □ No |

**Section 11: Exclusion Criteria Checklist**

| Criterion | Status |
| --- | --- |
| Hirschsprung's disease | □ Excluded □ Not applicable |
| Structural gastrointestinal anomalies | □ Excluded □ Not applicable |
| Irritable bowel syndrome (Rome IV) | □ Excluded □ Not applicable |
| Metabolic disorders (e.g., hypothyroidism) | □ Excluded □ Not applicable |
| Neurological impairments | □ Excluded □ Not applicable |
| History of gastrointestinal surgeries | □ Excluded □ Not applicable |
| Medications affecting GI motility | □ Excluded □ Not applicable |

**Section 12: Interviewer Information**

| Item | Information |
| --- | --- |
| Interviewer name | ......... |
| Interviewer qualification | □ Pediatric gastroenterologist □ Pediatric resident □ Other |
| Date of interview | ......... |
| Time taken for interview (minutes) | ......... |

**Confirmation**

I confirm that the information provided is accurate to the best of my knowledge and that the diagnosis was made based on Rome IV criteria.

**Parent/Guardian signature:** _________________ **Date:** _________

**Interviewer signature:** _________________ **Date:** _________
